# Implicit Processing of the Eyes and Mouth: Evidence from Human Electrophysiology

**DOI:** 10.1371/journal.pone.0147415

**Published:** 2016-01-20

**Authors:** Francesca Pesciarelli, Irene Leo, Michela Sarlo

**Affiliations:** 1 Department of Biomedical, Metabolic and Neurological Sciences, University of Modena and Reggio Emilia, Modena, Italy; 2 Department of Developmental Psychology, University of Padova, Padova, Italy; 3 Department of General Psychology, University of Padova, Padova, Italy; 4 Center for Cognitive Neuroscience, University of Padova, Padova, Italy; Bournemouth University, UNITED KINGDOM

## Abstract

The current study examined the time course of implicit processing of distinct facial features and the associate event-related potential (ERP) components. To this end, we used a masked priming paradigm to investigate implicit processing of the eyes and mouth in upright and inverted faces, using a prime duration of 33 ms. Two types of prime-target pairs were used: 1. congruent (e.g., open eyes only in both prime and target or open mouth only in both prime and target); 2. incongruent (e.g., open mouth only in prime and open eyes only in target or open eyes only in prime and open mouth only in target). The identity of the faces changed between prime and target. Participants pressed a button when the target face had the eyes open and another button when the target face had the mouth open. The behavioral results showed faster RTs for the eyes in upright faces than the eyes in inverted faces, the mouth in upright and inverted faces. Moreover they also revealed a congruent priming effect for the mouth in upright faces. The ERP findings showed a face orientation effect across all ERP components studied (P1, N1, N170, P2, N2, P3) starting at about 80 ms, and a congruency/priming effect on late components (P2, N2, P3), starting at about 150 ms. Crucially, the results showed that the orientation effect was driven by the eye region (N170, P2) and that the congruency effect started earlier (P2) for the eyes than for the mouth (N2). These findings mark the time course of the processing of internal facial features and provide further evidence that the eyes are automatically processed and that they are very salient facial features that strongly affect the amplitude, latency, and distribution of neural responses to faces.

## Introduction

The human face is the most important visual object we process everyday, providing information such as gender, race, age, emotional state, and identity. The ability to identify a face refers to the ability to discriminate among different exemplars of the face category, recognizing a face as familiar. This sophisticated competence is the result of the analysis of subtle differences between faces, which have a similar structure, where features (i.e., eyes, nose, mouth) are in a similar arrangement (i.e., two eyes above a nose, which is above a mouth). This ability is attributed to enhanced sensitivity to configural information in faces, as opposed to the analytic processing involved in object recognition (e.g., [1]). Relative to upright faces, recognizing inverted faces is surprisingly poor, and converging evidence proposes that this inversion effect is unique to faces and is due to the disruption of configural processing. Recently, some studies on adaption effects in relation to face perception seem to suggest that although inverted faces may not be processed configurally, they should share some processing mechanisms with upright faces (e.g., [2]).

Maurer et al. [1] have suggested that face processing involves several stages of configural processing: an initial stage that encodes the first-order relational information (two eyes above nose, nose above mouth), which is then combined into a second holistic stage that integrates facial features into a whole or a Gestalt; and a final stage that encodes the second-order relational information (which refers to fine spatial relations between features) which is essential for identifying individual faces.

Despite much research, the types of configural processing and the stages that underpin face perception are still under debate. In the past years several studies, using event-related potentials (ERPs), indirectly investigated the time course of face processing, but did not provide conclusive information about it ([3–7]; For an extensive and detailed description of the ERP components involved in face processing refer to our recent work, [8]). Recently we [8] have tried to shed light on the time course involved in face recognition, by investigating implicit face processing using ERPs in a masked priming paradigm in which the prime was presented for 33 ms. Based on our findings, we proposed that configural face processing can be processed unconsciously in the brain. In our study, several ERP components have been identified to manifest the processing of faces. In particular, the study showed two dissociable effects that emerged over time: An early effect (as reflected by P1, N1, P2, N170) indicating a fast perceptual processing, and a late effect (as reflected by N2 and P3), which may reflect identification and recognition processing. However, in our previous study we did not assess the influence of internal facial features in face processing.

In the past three decades, much research focused upon the importance of different features, such as the eyes, nose, and mouth. A hierarchy emerged in regard to the importance of such features, the eyes being more important than the mouth and nose [9–12]. However, the exact nature of the processing of facial features and its neural underpinnings are still under debate. We aimed to understand which face regions are predominantly used for face processing and the time course of their processing.

Studies using ERPs have identified a face-specific component called the N170. The N170 is a negative component peaking around 170 ms, distributed consistently over the posterior temporal regions, which is highly sensitive to faces (e.g., [10, 13, 14]). The N170 is assumed to reflect early perceptual face encoding stages [13, 15] in a holistic processing (i.e., gluing facial features together into a gestalt). The N170 is also sensitive to face inversion, which delays and alters its amplitude. The literature also suggests that the N170 is not affected by face familiarity [16]. Furthermore, it has been demonstrated that the N170 component is particularly sensitive to the eye region. In particular, it has been shown that the N170 is largest in response to whole faces and isolated eyes, and attenuated and delayed in response to other facial feature such as nose and mouth [10,17–19]. The N170 is also faster in latency when eyes are present within a face than when they are absent [20], and when the face does not contain eyes the inversion effect disappears [21]. It has been suggested that the inversion effect may be driven by the location of the eyes [18]. These findings suggest that the eyes play an important role in face processing and strongly affect the amplitude, latency, and distribution of neural responses to faces. It is worth noting that several authors have found no change in the N170 amplitude after eyes were removed from the face [20–22], supporting the view that the N170 reflects a holistic processing stage. However, Itier at al. [21] have proposed a neural face model where eye and face sensitive neuronal population are involved. Specifically, in this model it has been suggested that upright faces activate face-sensitive neurons, which would inhibit eye-sensitive neurons in the context of a configurally correct face. This would explain the lack of amplitude change with upright eyeless faces. However, presenting inverted faces (i.e., disrupting holistic processing), would stop this inhibition, and the N170 would reflect the co-activation of both neuronal populations. Both neuronal populations would also respond to isolated eyes. This would explain the larger N170 amplitude for eyes than for upright faces (see also [23]). Despite all this evidence, the exact function of the N170 is still a matter of debate.

To the best of our knowledge, only one other ERP component seems to be sensitive to the eyes region. Nemrodov, Anderson, Preston, and Itier [23], in a study where the authors investigated whether fixation on different facial features (forehead, nasion, left-eye, right-eye, nose, mouth) in intact and eyeless faces modulated early ERP components, found a larger P1, a positive component typically elicited by visual stimuli, for intact than eyeless faces when the fixation was on the eyes rather than on other facial features. This P1 modulation is explained by the authors as a difference in local contrast and might reflect the initial stages of the eye processing.

All these studies suggested that the eyes appear to have a salient role, however left many questions unanswered: 1. What is the time course of the processing of facial features? 2. What is their neural underpinning? 3. What are the ERP components involved in their processing? The aim of the present study is to shed light on all these open questions. In particular, given that the importance of the eyes and mouth information in the very first steps of face processing is also unclear, in the present study we performed new analyses on the dataset from our previous work [8]. By focusing on the implicit processing of distinct facial features, we aimed to address: 1. The temporal characteristics of eyes and mouth processing and the associated ERP components, and 2. Which features of a face (eyes or mouth) speed face detection. To this end, we used the methodology reported in Pesciarelli et al. ([8]; see also [24], Experiment 3). In particular, we used a masked priming paradigm in order to examine implicit processing of the eyes and mouth both in upright and inverted faces. We focused on these two features because they allowed us to use a comparable speed judgment (decide whether the mouth or eyes where open) in order to focus participants’ attention on these features of the face (to attribute two independent processing). Moreover, our choice is supported by several behavioral works showing that the most salient internal face features for face processing are, in order of importance, eyes, mouth, and nose [25, 26]. However, further research will be necessary in order to explore the neural mechanisms underlying other facial features, such as the nose. The masked priming paradigm is a strong methodological tool that allows to investigate the neural correlates of facial features processing prior to awareness and the early stages of face processing. To date, there are few and controversial studies that have investigated the different roles of the eyes and mouth in face processing and most of them have focused on the N170 component, so that little is known about the ERP markers of facial features. Moreover, no study has used a masked priming paradigm. Linking different ERP components to distinct face processing stages is essential to understand the mechanism of face perception. Thus, in the present study, we captured the brain temporal dynamics of implicit processing of the eyes and mouth on early (P1, N1, P2, N170) and late (N2, P3) ERP components. We expected to replicate the orientation effect on early components and the priming/congruency effect on late components shown in our previous work [8]. As a novel hypothesis, we expected that, if the eyes are the most relevant feature for face perception, an orientation and a priming/congruency effect would be obtained for the eyes even under implicit processing. In particular, we hypothesized to find ERP effects starting earlier for the eyes than for the mouth both for orientation and congruency.

## Method

### Ethics statement

The procedures have been approved by the Ethics Committee of the University of Padova.

### Participants

Fourteen students at the University of Padova (7 women), with an age ranging from 19 and 31 (mean = 20 yrs) participated in the experiment. All participants were right-handed, without a history of neurological or mental disorders and with normal or corrected-to-normal visual acuity. All participants were informed of their rights and gave written informed consent for participation in the study.

### Stimuli

Facial stimuli were taken from the NimStim Face Stimulus Set [27] and presented black and white. The background was black and the mean luminance was approximately the same for all pictures. The forward mask and prime were identical faces, while the target face was different from both the forward mask and prime. The prime was 25% smaller (visual angle 8.5°) than the forward mask and target (visual angle 11.3°) to avoid any apparent movement between the forward mask and prime stimuli.

The forward mask face had both eyes and mouth closed, the prime face had the eyes or mouth open. The target face had open mouth or open eyes, except on 50% of the trials in which both eyes and mouth were closed (catch trials). These catch trials were included to prevent response habituation, to control for attention and to make sure that the participants examined the whole face. Two types of prime-target pairs were used: 1. congruent (e.g., open eyes only in both prime and target or open mouth only in both prime and target) and 2. incongruent (e.g., open mouth only in prime and open eyes only in target or open eyes only in prime and open mouth only in target). Half of the trials were rotated by 180° to produce inverted mask, prime and target faces.

Each participant completed 640 trials (40 trials per condition), separated into four blocks, two blocks with upright faces and two blocks with inverted faces. It should be noted that the same two faces were used throughout the experiment. Face orientation was blocked whereas prime-target congruency was randomized within each block.

### Design and Procedure

**Fig 1:**
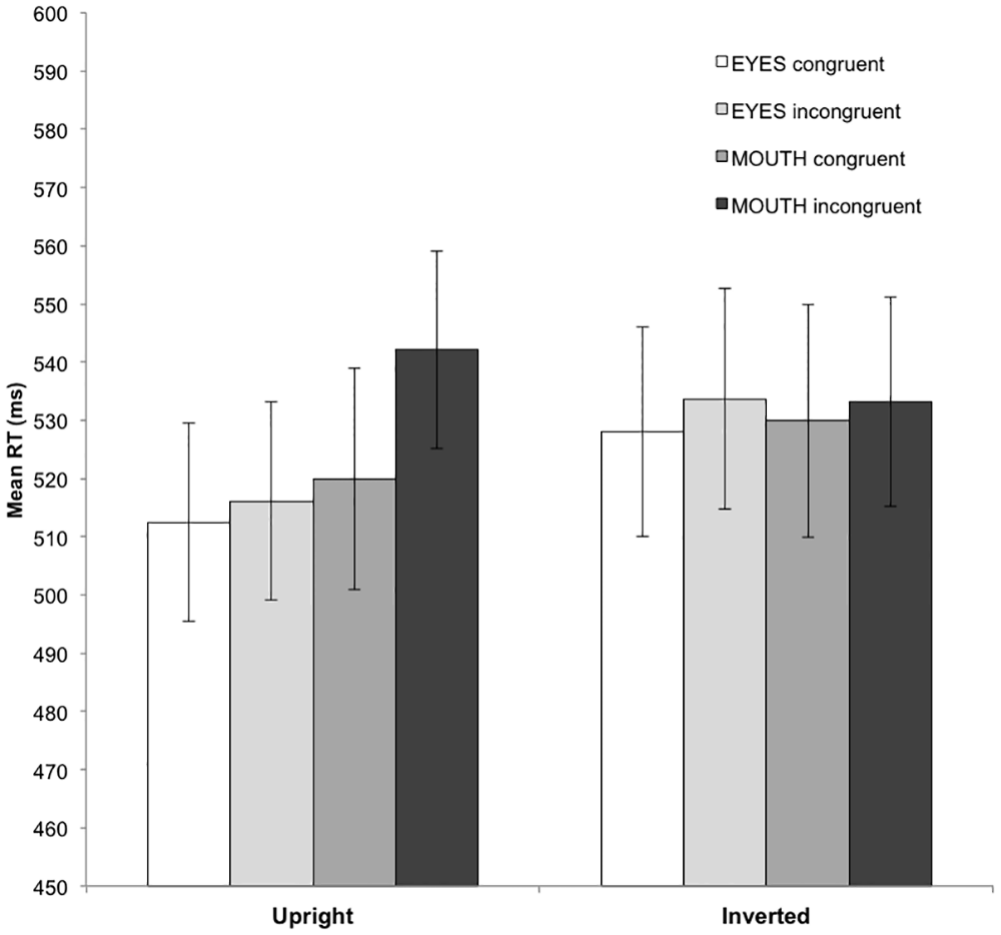
Behavioral results. Mean reaction times separately for upright and inverted faces in each experimental condition: open eyes congruent (white bar), open eyes incongruent (light grey bar), open mouth congruent (dark grey bar), and open mouth incongruent (black bar). Error bars represent standard errors.

An example of the stimulus presentation procedure is illustrated in Pesciarelli et al. ([18], Fig 1). All stimuli (faces) were displayed in the center of a CRT located approximately 100 cm directly in front of the participant. Each trial began with a fixation cross (+) in the middle of the screen. Five hundred milliseconds later, a 200-ms black screen that was replaced by a 1500-ms forward mask was presented. The forward mask was replaced at the same location on the screen by the prime item for 33 ms. The prime was then immediately replaced by the target, which remained onscreen until a response was made. Each response was followed by a 1000 ms blank screen.

Participants were instructed to press a response button as quickly and accurately as possible when the target face had the eyes open, another button when the target face had the mouth open and to withhold the response when the target face had neither eyes nor mouth open (catch trials). This speed judgment for the eyes and mouth on target stimulus allowed focusing participants’ attention on these features of the face (to attribute two independent processing of the eyes and mouth). It is worthy of note that the eyes and mouth were never simultaneously open in both the prime and the target face. At the end of the experiment, participants were asked to describe what they perceived between the forward mask and target. They were then informed of the presence of the prime and were asked if they could describe the face. None of the participants explicitly reported that the prime face was the same as the forward mask face. Thus, participants were unable to identify the prime.

### EEG recording and analysis

The electroencephalogram (EEG) was recorded from 19 tin electrodes mounted in an elastic cap according to the International 10–20 System [28] at sites FP1, FP2, F3, Fz, F4, F7, F8, C3, Cz, C4, T3, T4, T5, T6, P3, Pz, P4, O1, O2 and right mastoid. The signals were recorded using a left mastoid reference, and then re-referenced off-line to the average of the left and right mastoids. For the purpose of artifact scoring, vertical and horizontal electro-oculograms (EOGs) were recorded. Electrode pairs (bipolar) were placed at the supra- and suborbit of the right eye and at the external canthi of the eyes. All electrode impedances were kept below 10 kΩ. The EEG and EOG signals were amplified with Neuroscan Synamps (El Paso, TX, USA), bandpass filtered (0.1–70 Hz), digitized at 500 Hz (16 bit AD converter, accuracy 0.08 uV/bit) and stored on a Pentium IV computer.

Continuous EEG data were corrected for eyeblinks using a regression-based correction algorithm (Scan 4.1 software). The EEG was then segmented off-line into 900-ms epochs from 100 ms before to 800 ms after target onset. The EEG epochs were baseline-corrected against the mean voltage during the 100-ms prestimulus period. All EEG epochs were visually scored for eye movement and other artifacts, and each portion of data containing artifacts greater than ±70 uV in any channel was rejected for all the recorded channels prior to further analysis. Artifact-free trials with correct behavioral responses were separately averaged for each subject in each experimental condition.

Based on visual inspection of grand average ERP waveforms and in line with previous literature, the following components were identified for target onset at frontal (F3, Fz, F4), central (C3, Cz, C4) and parietal (P3, Pz, P4) scalp sites: N1 from 60 to 140 ms after target onset; P2 from 120 to 210 ms after target onset; N2 from 165 to 265 ms after target onset and P3, from 350 to 510 ms after target onset. At posterior electrode sites (T5, T6, O1, O2), the following ERP components were considered: P1 from 40 to 100 ms and N170 from 100 and 180 ms after target onset. For each ERP component amplitude was measured as mean activity within the respective time window.

### Statistical analysis

For each participant, outlier correction [29] on RTs was applied (M 1.39% rejected trials). Behavioral and ERP analyses were carried out only on trials with correct responses (M 98% of correct trials). Catch trials were excluded from the analyses.

The mean response times (RTs) of correct responses were submitted to separate analyses of variance (ANOVAs) that considered face orientation (upright, inverted), prime-target congruency (congruent, incongruent), and internal features (eyes open, mouth open) as within-subject factors. Hit rates were not formally analyzed because of ceiling effects, with all conditions averaging 98% correct.

ERP effects time-locked to the onset of the target face were evaluated taking into account 6 clusters of electrodes representing the mean amplitude of three electrodes in close position: Anterior (F3, Fz, F4), Central (C3, Cz, C4), Posterior (P3, Pz, P4), Left (F3, C3, P3), Midline (Fz, Cz, Pz), Right (F4, C4, P4). ANOVAs were conducted on mean ERP amplitudes with face orientation (upright, inverted), prime-target congruency (congruent, incongruent), internal features (eyes open, mouth open), longitude (anterior, central, posterior) and latitude (left, midline, right) as within-subjects factors. At posterior electrode sites, the ANOVA included the following within-subject factors: face orientation, prime-target congruency, internal features and EEG site (T5, T6, O1, O2).

Post-hoc mean comparisons (Newman-Keuls) were employed to further examine significant effects (using a p < .05 criterion for significance). When appropriate, degrees of freedom were adjusted according to the method of Greenhouse—Geisser, and only corrected significance levels are reported. The level of significance testing was *p* = .05. Below, we discuss only those interactions that are of interest to our study.

## Results

### Behavior

The RTs to correct answers in the different conditions are plotted in Fig 1.

The three-way within-subject ANOVA conducted on the RT data yielded a significant main effect of prime-target congruency [F(1,13) = 10.80, p < .01, η_p_^2^ = .45, observed power = .86], with faster RTs for congruent than incongruent trials. There was no significant main effect of face orientation and internal features [F(1,13) = 1.4, p > .26, η_p_^2^ = .09, observed power = .19; F(1,13) = 1.11, p > .31, η_p_^2^ = .08, observed power = .16, respectively]. Moreover, the analysis revealed a marginally reliable orientation by internal features interaction [F(1,13) = 4,14, p < .06, η_p_^2^ = .24, observed power = .47], post-hoc comparisons showed faster RTs for the eyes in upright faces than for the eyes in inverted faces [p < .01], the mouth in upright faces [p < .03] and the mouth in inverted faces [p < .04]. Furthermore, a reliable orientation by congruency by internal features interactions was observed F(1,13) = 6.05, p < .03, η_p_^2^ = .32, observed power = .62]. To further explore this three-way interaction, separate analyses for internal features (open eyes and open mouth) were conducted. Open eyes: The analyses revealed a marginally significant orientation effect [F(1,13) = 4.01, p < .06, η_p_^2^ = .24, observed power = .46], with faster RTs for upright than inverted faces. No other effects reached significance (*ps* > .1). Open mouth: The analyses showed a congruency priming effect and an orientation by congruency interaction [F(1,13) = 6.15, p < .03, η_p_^2^ = .32, observed power = .63; F(1,13) = 5.74, p < .03, η_p_^2^ = .31, observed power = .61, respectively]. This latter interaction indicates a congruency priming effect, with slower RTs for incongruent than congruent trials (p < .01), but just for upright faces.

Moreover, the RT distributions were tested for assumptions of normality using the Shapiro-Wilks test and were found to be not significantly different from a normal distribution (all ps > .14).

### Event-Related Potentials

**Fig 2:**
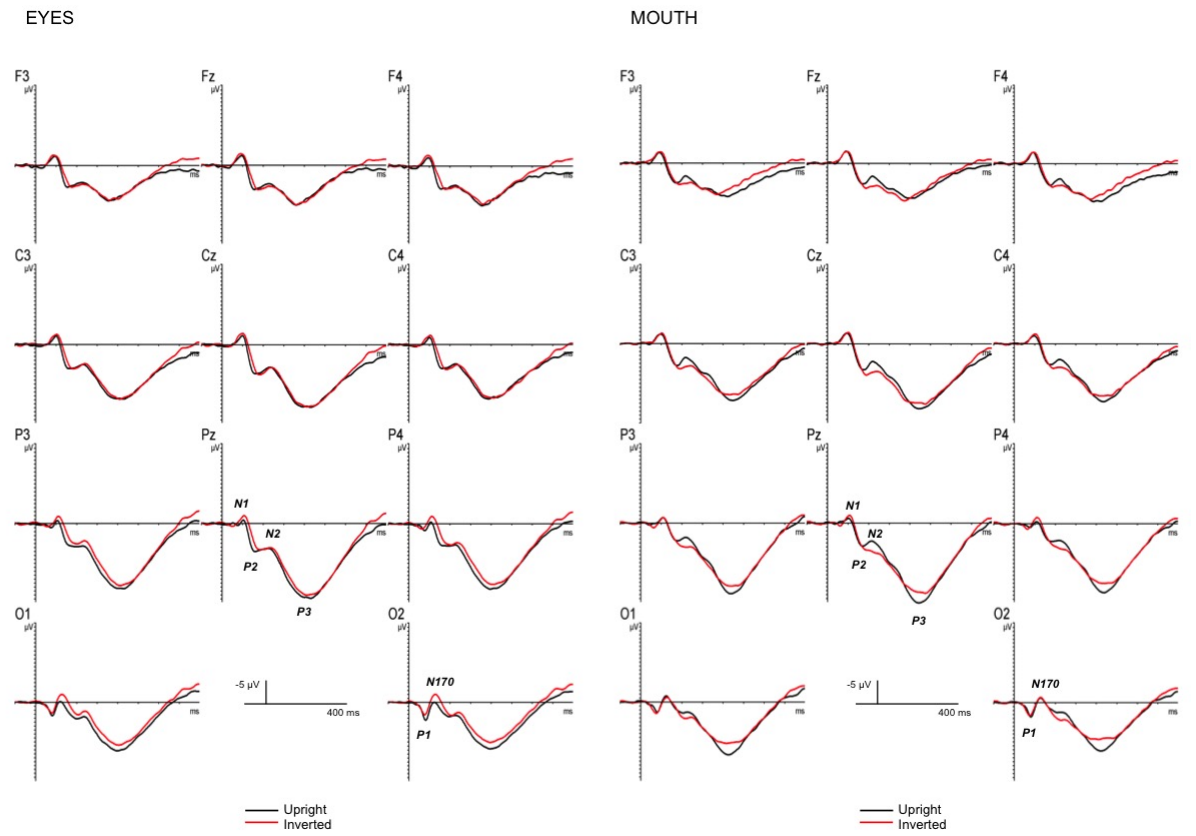
ERP effects of face orientation. Grand-averaged event-related potentials to target faces as a function of internal features and face orientation.

**Fig 3:**
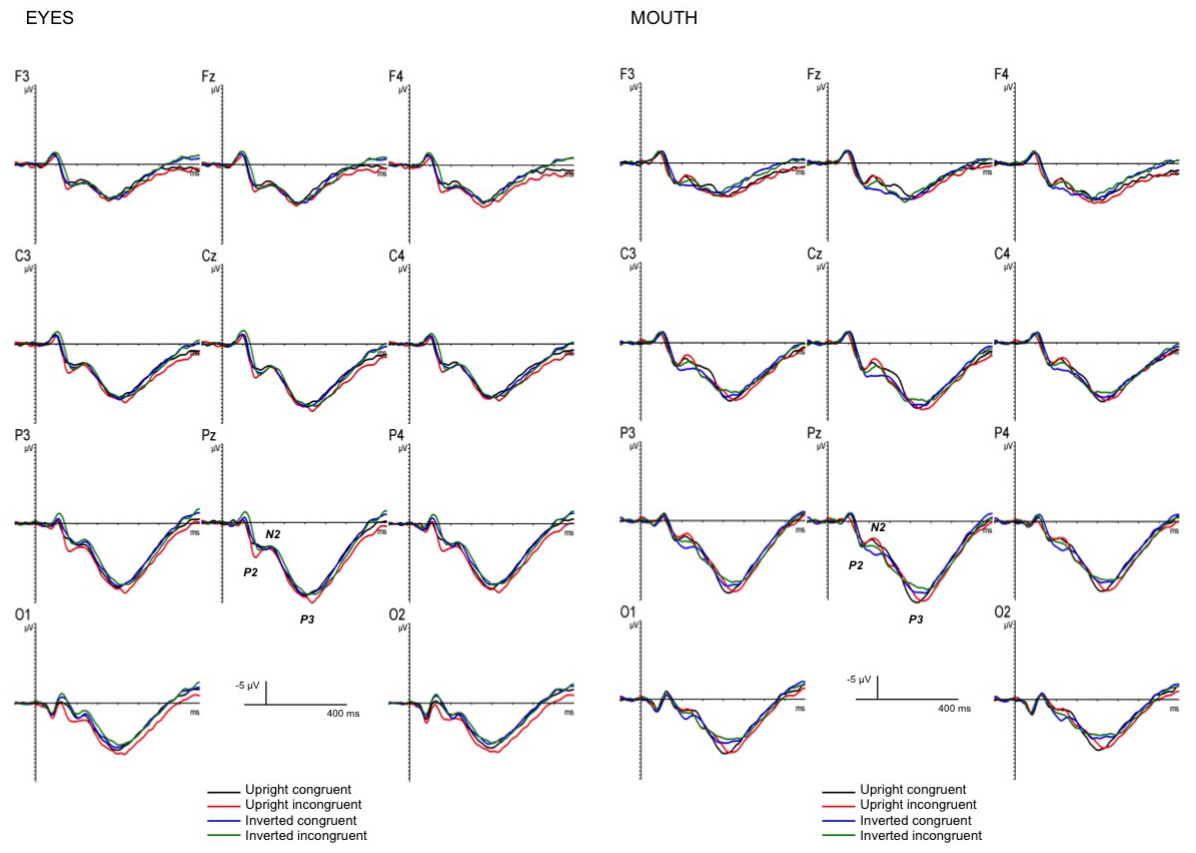
ERP effects of masked priming. Grand-averaged event-related potentials as a function of prime-target congruency, internal features and face orientation.

**Fig 4:**
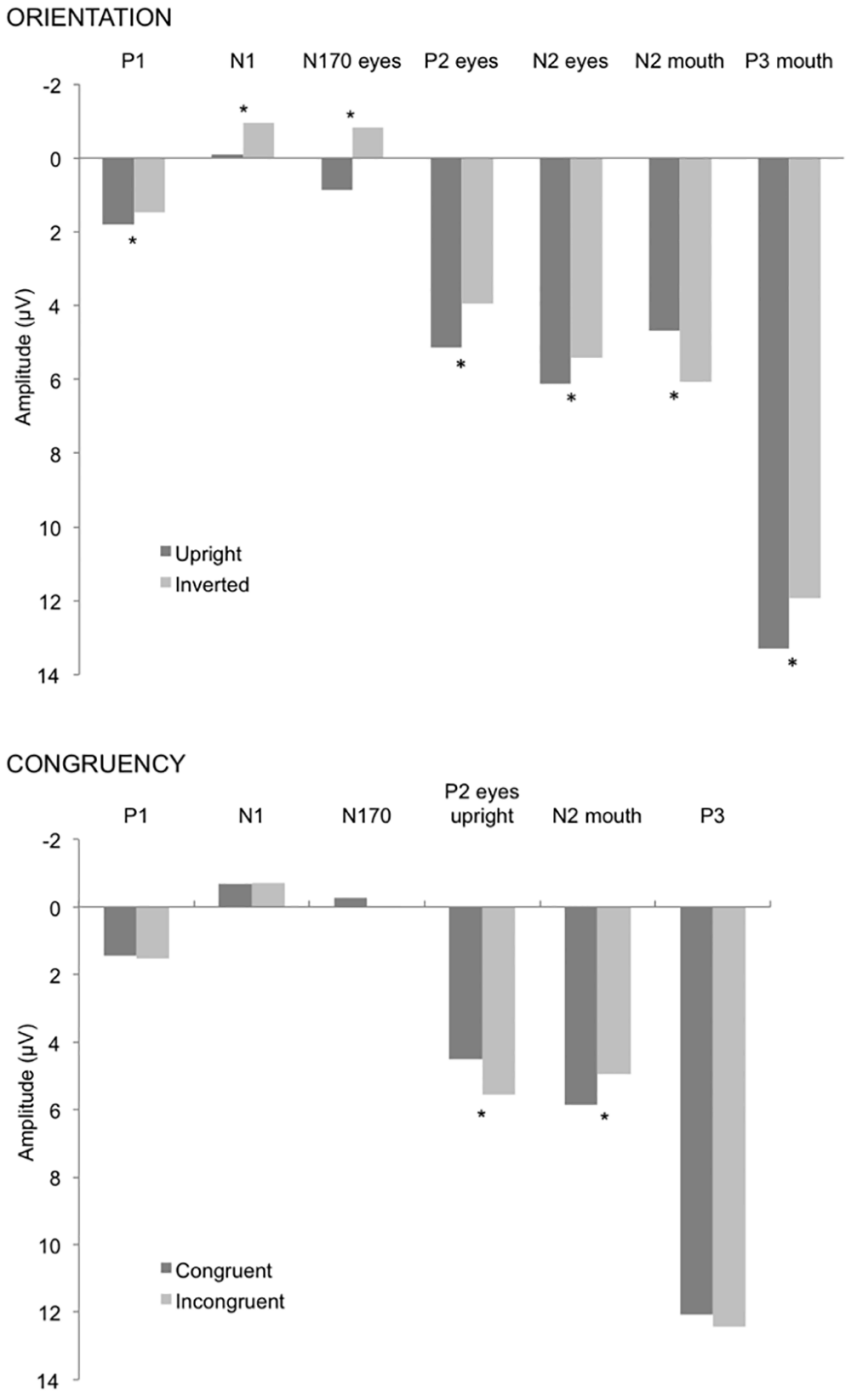
Time course of implicit face processing. Histogram of the amplitude of face-sensitive ERP components as a function of internal features and face orientation (upper panel) and as a function of internal features and prime-target congruency (lower panel). *p < 0.05.

Grand-averaged ERPs elicited by target faces are represented in Fig 2 as a function of internal features and face orientation, and in Fig 3 as a function of prime-target congruency, internal features and face orientation. Histogram of the amplitude of all ERP component effects as a function of internal features, prime-target congruency and face orientation is represented in Fig 4.

#### P1

The ANOVA revealed a significant main effect of EEG site [F(3,39) = 10.28, p < .0001, η_p_^2^ = .44, observed power = .99], indicating a larger P1 in the occipital than occipito-temporal area, and a significant face orientation by EEG site interaction [F(3,39) = 3.49, p < .02, η_p_^2^ = .21, observed power = .74], revealing a larger P1 amplitude for upright than inverted faces only in the right hemisphere (*ps* < .01).

#### N1

The analysis on the N1 range showed reliable main effects of longitude [F(2,26) = 11.67, p < .001, η_p_^2^ = .47, observed power = .99], and latitude [F(2,26) = 3.61, p < .04, η_p_^2^ = .22, observed power = .62]. Overall, the N1 amplitude was larger in the fronto-central than parietal regions and on the left and midline than on the right sites. The significant face orientation by latitude interaction [F(2,26) = 3.73, p < .04, η_p_^2^ = .22, observed power = .63] showed that the N1 amplitude was larger for inverted than upright faces on the right (*p*s < .001) than on the left and midline sites.

#### N170

The ANOVA on the N170 face-specific component showed a significant main effect of face orientation [F(1,13) = 4.8, p < .05, η_p_^2^ = .27, observed power = .53], showing larger amplitudes for inverted than upright faces. Moreover, the analysis revealed two significant interactions, one between face orientation and internal features [F(1,13) = 6.85, p < .02, η_p_^2^ = .35, observed power = .68], and the other between face orientation, internal features, and EEG sites [F(3,39) = 3.47, p < .03, η_p_^2^ = .21, observed power = .73]. The former interaction suggests a reliable orientation effect for the eyes (p < .01), with a larger N170 for inverted than upright faces, but not for the mouth, and a larger N170 for the mouth relative to the eyes, but only in the upright face (p < .02). The latter interaction shows that the orientation effect for the eyes was larger in the occipital than in the occipito-temporal sites (*ps* < .0001).

#### P2

The analysis showed three significant interactions: 1. Longitude by latitude [F(4,52) = 5.46, p < .001, η_p_^2^ = .3, observed power = .96], indicating a larger P2 in the midline centro-parietal sites (ps < .04); 2. Longitude by orientation by internal features [F(2,26) = 4.29, p < .03, η_p_^2^ = .25, observed power = .7], showing an orientation effect only for the eyes, with a larger P2 for upright than inverted faces in the centro-parietal region (ps < .02); 3. orientation by internal features by congruency [F(1,13) = 8.36, p < .01, η_p_^2^ = .39, observed power = .76], indicating a congruency effect only for the eyes and only in upright faces (*ps* < .02), with a larger P2 for incongruent than congruent trials.

#### N2

The ANOVA on the N2 revealed two reliable interactions, one between internal features and congruency [F(1,13) = 11.01, p < .01, η_p_^2^ = .46, observed power = .87], suggesting a congruency effect for the mouth, but not for the eyes, with a larger N2 for incongruent than congruent trials and a larger N2 for the incongruent condition for the mouth relative to the incongruent condition for the eyes (*ps* < .02), and the other between longitude, orientation and internal features [F(2,26) = 5.73, p < .01, η_p_^2^ = .31, observed power = .82], reflecting an orientation effect in all three regions for the mouth and only in the parietal region for the eyes (*ps* < .03), with a larger N2 for upright than inverted faces for the mouth and with a larger N2 for inverted than upright faces for the eyes.

#### P3

Amplitude: The ANOVA revealed a significant longitude by latitude and a reliable orientation by internal features interactions [F(4,52) = 10.83, p < .00001, η_p_^2^ = .45, observed power = .1; [F(1,13) = 7.6, p < .02, η_p_^2^ = .37, observed power = .72, respectively]. The first interaction indicates a larger P3 in the midline parietal area, while the latter indicates an orientation effect for the mouth, with larger P3 amplitude for the upright than inverted faces and a larger P3 for the mouth open than the eyes open in the upright faces (*ps* < .01).

Latency: Visual inspection of the P3 time-window suggested a latency effect, thus an ANOVA considering the same factors was carried out on the P3 peak latency, which was extracted as the point in time at which the largest positive value was detected in a 350–510 ms time window. The analysis on the P3 latency showed a significant main effect of orientation [F(1,13) = 5.02, p < .04, η_p_^2^ = .28, observed power = .55], revealing an increasing latency for the upright relative to the inverted face; and a significant main effect of internal features [F(1,13) = 12.5, p < .01, η_p_^2^ = .49, observed power = .9], showing an increasing latency for the mouth open relative to the eyes open faces. Moreover, the ANOVA revealed a significant longitude by orientation by congruency interaction [F(2,26) = 3.54, p < .04, η_p_^2^ = .21, observed power = .61], revealing an increasing latency for incongruent than congruent trials in upright faces only, and more pronounced in the centro-parietal region (*ps* < .02).

## Discussion

In the present work we investigated the time-course of implicit facial features processing. In particular we explored face-sensitive ERP components and their time course by comparing ERPs elicited by distinct internal facial features, such as eyes and mouth, of upright and inverted faces. Participants were exposed to a masked priming paradigm in which prime-target pairs were congruent or incongruent. Priming was inferred from both reaction times for congruent versus incongruent targets, and ERP amplitude differences at various latencies post-target onset for the same comparison. Different feature-sensitive modulations of ERPs are likely to reflect different stages of face processing from the perceptual analysis and structural encoding of face components up to the identification and recognition of face stimuli. Behavioral and ERPs findings revealed a consistent pattern of results.

On the behavioral side, we found a congruency/priming effect for upright but not for inverted faces. We did not replicate the priming effect for inverted faces showed in Williams et al. [24]. See Pesciarelli et al. [8] for a possible explanation. Moreover, our behavioral results showed faster RTs for the eyes in upright faces than the eyes in inverted faces, the mouth in upright and inverted faces. Our evidence is consistent with a recent study showing that the eyes are important cue for face perception [30]. Specifically, Hills et al. [30] suggest that we do not normally fixate enough upon the eyes in inverted faces and that this may cause a delay in the accurate processing of the face. In addition, Hills et al.’s data seem to suggest that multiple mechanisms are involved in inversion effects, with different task or paradigms recruiting different mechanisms. Interestingly, our findings also showed a congruency priming effect for the mouth, but only in upright faces. This latter result likely reflects an inhibitory effect (slower reaction times on the incongruent condition) mediated by the eyes-open in the prime stimulus, such that the feature attended to in the target stimulus was the eyes open (congruent) and not the mouth open (incongruent). These data support the idea of a special sensitivity to eyes rather than to mouth. The eyes and mouth seem to engage different cognitive mechanisms, and this difference, as we will discuss in the next sections, is also reflected in qualitatively different accompanying ERP modulations.

### Internal feature ERP effects of face orientation

Although no effect of face orientation was found behaviorally, such an effect was observed in all the analyzed ERP components (P1, N1, N170, P2, N2, P3), starting at about 80 ms, as already reported in our previous study [8]. For an extended discussion of the implication of each component on the orientation effect see Pesciarelli et al. [17]. As a novel feature of the present study, we were primarily interested in examining whether different internal features might specifically influence these orientation effects. Crucially, our results showed a N170 and P2 orientation effect elicited by the eyes but not by the mouth, a N2 orientation effect elicited by both the eyes and mouth, and a P3 orientation effect elicited only by the mouth.

It is interesting to note that in our study the first component affected by eye processing was the N170, with larger amplitude for inverted then upright faces. This component is elicited primarily by face stimuli and in specific by the detection and analysis of eyes (e.g., [20]). Therefore, the sensitivity of the N170 to the eye region, found in our work, is in line with previous studies (e.g., [10, 20]) and seems to confirm the assumption that the N170 orientation sensitivity is at least partially dependent on the presence of the eye region [17]. This sensitivity for the eyes was also found for the P2 component. The P2 was more positive for upright compared to inverted faces, but only for the eyes. What processes the P2 indexes are a matter of debate; however, our findings are in accordance with the suggestion that this component is sensitive to facial configuration [31,32]. Face orientation also affected the N2, with a larger negativity for upright than inverted faces for the mouth and with a larger negativity for inverted than upright faces for the eyes. This effect on the N2 agrees with previous ERP evidence suggesting that the N2 component is modulated consistently by face stimuli [33–35] and adds a new evidence of its sensitivity to feature processing. It is interesting to note that the N2 orientation effect for eyes and mouth goes in opposite directions. One possible explanation is that the N2 is the only component to be affected by both eyes and mouth processing and this N2 time-window seems to index the temporal point at which the eye processing is at its ending while the mouth processing at its beginning, suggesting different mechanisms underlying eye and mouth processing. Since there is not previous research looking that at N2 responses to just mouths, our interpretation has a speculative nature and needs to be examined in future studies. The other component affected by face orientation was the P3, with larger amplitude for upright than inverted faces, but only for the mouth. This component seems to reflect the processes of memory access and attentional resource allocation (e.g., [36]) evoked by the encoding of the mouth in target stimuli.

These patterns of results show, firstly, an important role of the eyes in early face encoding, starting at around 150 ms, and secondly, distinguishable stages involved in featural face processing. In particular, these findings suggest the existence of two different stages of feature processing: one starting early (about 150 ms, indexed by the N170 onset) in which the first feature to be processed is the eyes, and one starting later (about 200 ms, indexed by the N2 onset) in which the other features start to be processed, in our case the mouth. This suggestion is in line with Nemrodov et al.’s [23] model, in which they propose not only the existence of face-sensitive and eye-sensitive neurons, as in Itier et al. [21], but also the presence of several different populations of neurons, one responding to the eyes, one to the nose, one to the mouth, one to the ears, and so on, where the eyes are the anchor point from which the position and orientation of other facial features are encoded on the basis of a reference model of an upright human face. Thus, it is not surprising that, as suggested by our results, the eyes are the first facial feature to be processed during the time course of face encoding.

### Internal feature ERP effects of masked priming

We found a congruency/priming effect on ERP components such as the P2, N2 and P3. These patterns of results indicate a congruency effect starting earlier for the eyes (around 150 ms) and later for the mouth (around 200 ms). In our study, the P2 is the first component to be affected by congruency and crucially this effect is only present on the eyes and only in upright faces, with a larger positivity for incongruent than congruent trials. This P2 modulation is not surprising if we consider that this component has been suggested to be sensitive to facial configuration [31,32]. The other component affected by priming was the N2, with a larger negativity for incongruent than congruent trials for the mouth in both upright and inverted faces. The results also showed a larger N2 for the incongruent condition for the mouth relative to the incongruent condition for the eyes. This latter effect likely reflects the inhibitory behavioral effect (slower reaction times on the incongruent condition for the mouth) discussed above. It is worthy of note that this component marks the temporal point at which the mouth starts to be encoded. This N2 modulation suggests an automatic neural mechanism underlying the recognition and identification of faces and seems in accordance with previous neurophysiological studies that consider the N2 component to be a reflection of an automatic, non-conscious attention-orienting response [34]. The last component affected by congruency/priming is the P3. An increased P3 latency was found for incongruent than congruent trials in upright faces only. Interestingly, an increasing latency for the mouth-open relative to the eyes-open faces was found. This shorter P3 latency observed for the eyes might indicate facilitated encoding and retrieval of stimuli that matched an active memory representation.

It is interesting to note that, in our experiment, the N170 in both upright and inverted faces was not modulated by face congruency. This result is in line with previous works [13,16,8] in which it has been demonstrated that the N170 is not affected by familiarity. Eimer [13] suggested that this component reflects processes prior to the recognition and identification of individual faces. Thus, the N170 seems to reflect the perceptual encoding of face components rather than the processing stages involved in face identification.

To note that our findings did not replicate the P1 modulation for the eyes reported by Nemrodov et al. [23]. One likely explanation for this discrepancy is methodological: in the present experiment we used a design where the face stimuli were masked and this implicit presentation may have caused the lack of the P1 effect.

There is a limitation in our study that needs to be addressed. In the present work a small sample-size (14 participants) has been employed and this may have limited the potential for detecting other possible effects. Nevertheless, our results would still be relevant insofar as they show a relevant difference in the time-course of internal facial features.

## Conclusion

While sharing the main dataset, the current study importantly extends our previous work [8] by providing detailed information on the time course of implicit processing of specific facial features. Taken together, our findings suggest that face orientation is initially processed in an automatic mode using the first-order information, where after the first 150 ms (at N170 onset) the effect is driven by the eyes and then (at N2 onset) by the other facial features (the mouth). After the first 200 ms, the face processing system is affected by congruency/priming and creates an upright face representation using the second-order relational information, starting first by processing the eyes (at P2 onset) and then (at N2 onset) the other facial features (the mouth). However, an alternative account suggests that second-order configural processing is not important for face perception (e.g., [2]). Some research suggests the importance of a feature-based model that builds a representation from subregions of the face [37]. More specifically, the processing of individual featural information, based on feature saliency, seems to be crucial for face representation.

To conclude, the present work has marked the time-course of the processing of internal facial features, such as the eyes and mouth, with the eyes being a very silent element of face processing. Further research is necessary to better explore the neural mechanisms underlying facial feature processing.
